# Myosin modulators: emerging approaches for the treatment of cardiomyopathies and heart failure

**DOI:** 10.1172/JCI148557

**Published:** 2022-03-01

**Authors:** Sharlene M. Day, Jil C. Tardiff, E. Michael Ostap

**Affiliations:** 1 Division of Cardiovascular Medicine, University of Pennsylvania, Philadelphia, Pennsylvania, USA.; 2 Department of Biomedical Engineering, University of Arizona, Tucson, Arizona, USA.; 3 Pennsylvania Muscle Institute and Department of Physiology, University of Pennsylvania, Philadelphia, Pennsylvania, USA.

## Abstract

Myosin modulators are a novel class of pharmaceutical agents that are being developed to treat patients with a range of cardiomyopathies. The therapeutic goal of these drugs is to target cardiac myosins directly to modulate contractility and cardiac power output to alleviate symptoms that lead to heart failure and arrhythmias, without altering calcium signaling. In this Review, we discuss two classes of drugs that have been developed to either activate (omecamtiv mecarbil) or inhibit (mavacamten) cardiac contractility by binding to **β**-cardiac myosin (MYH7). We discuss progress in understanding the mechanisms by which the drugs alter myosin mechanochemistry, and we provide an appraisal of the results from clinical trials of these drugs, with consideration for the importance of disease heterogeneity and genetic etiology for predicting treatment benefit.

Myosin modulators are a novel class of pharmaceutical agents that were recently developed to treat patients with cardiomyopathies. Dilated cardiomyopathy (DCM) and hypertrophic cardiomyopathy (HCM) are the two most common subtypes — both inheritable, progressive conditions that often lead to heart failure and arrhythmias. A dilated left ventricular (LV) chamber and impaired contractility are pathophysiological hallmarks of DCM, while a smaller LV cavity and “supranormal” ejection fraction with impaired diastolic dysfunction are characteristic of HCM. These opposing effects on contractile function are, in part, explained by the divergent effects of causal genetic variants on sarcomeric function that act as the inciting triggers for adverse cardiac remodeling.

Positive inotropic therapy for DCM has been a long-sought-after therapeutic goal. Adrenergic agonists and phosphodiesterase inhibitors are currently used for acute management of decompensated heart failure, as palliative agents, or as a bridge to heart transplant in very advanced disease (1). However, these agents potentiate arrhythmias and increase mortality, likely related to increased oxygen demand and increased intracellular calcium levels, limiting their scope for long-term use (2, 3). Calcium sensitizers have also failed to show a treatment benefit in chronic heart failure and appear to have proarrhythmic properties (4). In theory, direct and selective activation of the actin and myosin interaction could enhance contractility without these untoward side effects. A high-throughput screen of approximately 40,000 small molecules that used a kinetic readout of myosin adenosine triphosphatase (ATPase) activity identified CK1827452/AMG-423 (omecamtiv mecarbil, Cytokinetics) as the leading compound that increased ATPase activity in a Ca2+-regulated actin-activated myosin ATPase assay (5, 6). A related compound, MYK-491 (danicamtiv), was subsequently developed using a similar high-throughput screening approach (7). Both compounds enhance contractility without inducing changes in the calcium transient, and clinical trials have shown treatment efficacy for patients with systolic heart failure of varying etiologies. The proportion of patients with genetic DCM in these studies is unknown.

Conversely, a strategy to decrease the myosin ATPase rate is being explored as a treatment for patients with HCM. A high-throughput screen identified MYK-461 (mavacamten, MyoKardia — now Bristol Myers Squibb) as a compound that decreases myosin ATPase activity, acting as an allosteric modulator to stabilize the autoinhibited state of cardiac myosin (8, 9). Another allosteric myosin modulator with similar properties, CK3773274/CK-274 (aficamten, Cytokinetics), was more recently developed (10). These modulators have shown clinical efficacy for the treatment of HCM and are in various stages of clinical trials.

Myosin modulators are commonly categorized as either “myosin activators” (omecamtiv, danicamtiv) or “myosin inhibitors” (mavacamten, aficamten). But this description is oversimplified and in some aspects misleading. This Review will delve in depth into the mechanisms of action of myosin modulators, some of which differ from or are even inconsistent with the originally proposed models. An appraisal of the results from clinical trials of these drugs will be provided, and consideration for the importance of disease heterogeneity and genetic etiology for predicting treatment benefit will be discussed.

## The myosin ATPase duty cycle

**Figure 1:**
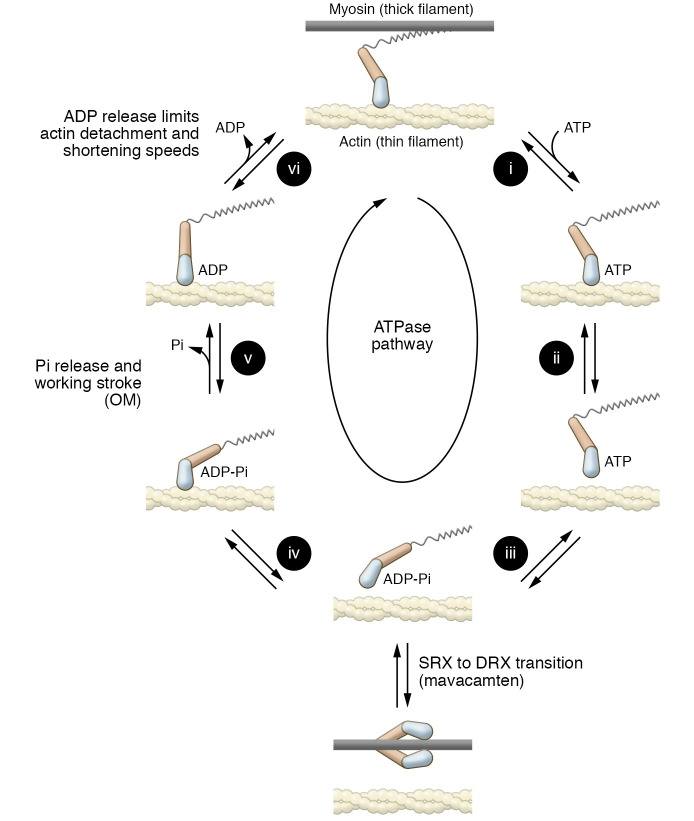
Myosin mechanochemical cycle. The relationship of the biochemical and conformational states of cardiac myosin is shown as the motor progresses through the ATPase cycle. For clarity, myosin molecules are illustrated with single motor domains. The force-generating working stroke occurs with phosphate release (step v). OM was originally developed to increase the rate of this transition, but subsequent studies have shown that it also affects the working stroke and subsequent kinetic steps. Danicamtiv likely works by the same mechanism, but biochemical analyses have not been performed to confirm this. The SRX-to-DRX transition is shown as an off-ATPase-pathway transition. Mavacamten is thought to stabilize the SRX state (see text and Figure 3), and presumably aficamten acts similarly in this regard. Adapted with permission from *Biophysical Journal* (12).

Understanding the mechanistic rationale for the development of myosin modulators requires knowledge of the relationship between mechanical force and the intermediates in the myosin ATPase pathway (Figure 1 and ref. 11). Briefly, myosin detaches from actin after ATP binding (steps 1 and 2, Figure 1), where it isomerizes to tilt its lever arm to the pre–power stroke conformation and hydrolyzes ATP to ADP and inorganic phosphate (Pi). The hydrolysis products remain noncovalently bound to the active site (step 3, Figure 1). Myosin rebinds actin, and the lever arm tilts toward the barbed end of the actin filament (i.e., toward the sarcomere Z-disk), resulting in the force-generating working stroke, phosphate release, and sarcomere shortening (steps 4 and 5, Figure 1). The transition that accompanies Pi release results in myosins that are tightly bound to actin and bear force (actomyosins [AMs]). The magnitude of force-generating myosins in the sarcomere depends directly on the fraction of the ATPase cycle time in which myosin is in the AM-ADP state (12). ADP is then released from the AM-ADP complex to complete the cycle (step 6, Figure 1). It is this ADP release step that limits the speed of sarcomere shortening (13).

## Rationale for development of omecamtiv mecarbil

It was the goal of the developers of omecamtiv mecarbil (OM) to find a small molecule that specifically binds β-cardiac myosin (MYH7) and activates Ca2+-regulated actin-activated myosin ATPase activity. From a high-throughput screen, a small molecule was found that increases the rate of phosphate release (step 5, Figure 1; and ref. 5). The proposed kinetic modification of myosin was expected to increase the fraction of cycle time in which myosins are bound to actin in a force-bearing state (i.e., increasing the duty ratio), resulting in higher force production from an ensemble of myosins (Fens) without affecting the rate of shortening (5). Additionally, an important goal was to achieve myocyte activation without affecting the calcium-release and calcium-uptake machinery, which can lead to proarrhythmic effects (5).

The original OM publication showed convincingly that the drug increases the rate of phosphate release from the AM-ADP-Pi state (step 5, Figure 1; and ref. 5), which was confirmed by subsequent publications (14–16). Additionally, the goal of activating muscle without affecting calcium release kinetics was achieved at low concentrations of OM (5). Structural and biochemical investigations provided biophysical details about the binding of OM to the M-ADP-Pi state (17), with additional studies showing binding to the apo state (18). Nevertheless, reports soon established that OM binding has properties inconsistent with myosin activation (14, 15, 19). Strikingly, OM inhibits actin gliding in the in vitro motility assay at all OM concentrations tested (18, 20). Biochemical experiments have also revealed that OM affects kinetic steps other than Pi release that result in a decrease in actin-activated ATPase activity of recombinant cardiac myosin when interacting with purified porcine cardiac thin filaments at saturating OM and calcium concentrations (14). Spectroscopic and modeling studies suggested that OM affects the kinetics of the structural changes that accompany the power stroke (15, 21). Finally, physiological experiments showed that micromolar concentrations of OM affect tension development and relaxation in myocytes and muscle fibers (22–27). These phenomena are inconsistent with the originally proposed model for OM activation of myosin in muscle (5).

## OM is a myosin inhibitor and muscle activator

To determine the effect of OM on myosin mechanochemistry, optical trapping studies were performed with recombinant human MYH7 (28, 29). Optical trapping is a biophysical technique that allows interrogation of the functional behavior of single myosin molecules, including the working stroke size, unitary force production (Funi), and actin-attachment lifetime during active ATPase cycling (30, 31). An advantage of this single-molecule technique is that the unitary behaviors of the motors are observed, without the need to infer their properties from averages obtained from large ensembles.

If OM functions as originally proposed, the entry of myosin into the force-bearing states would be increased, while the myosin working stroke size, actin-attachment lifetime, and force-dependent kinetics would be unchanged by drug binding. Surprisingly, it was found that OM potently inhibits the size of the myosin working stroke more than 10-fold, decreasing it from 5.4 nm to less than 0.4 nm at saturating concentrations. It was also found that the actomyosin attachment duration is prolonged 5-fold at physiological ATP concentrations, and the detachment rate of myosin from actin becomes independent of both ATP concentration and force applied to the myosin.

These studies (28), and those described above, make it clear that OM is not an activator of myosin, but counterintuitively a myosin inhibitor. These properties appear inconsistent with a drug that improves myocardial function. Nevertheless, experimental and clinical studies show that OM is a cardiac muscle activator at therapeutic concentrations (5, 32–34). Clearly, binding of a small molecule to myosin that inhibits myosin activity is not consistent with a motor-activated model of action. Thus, the originally described mechanism for this drug must be revised.

## The SEPTA model for OM activation of muscle

**Figure 2:**
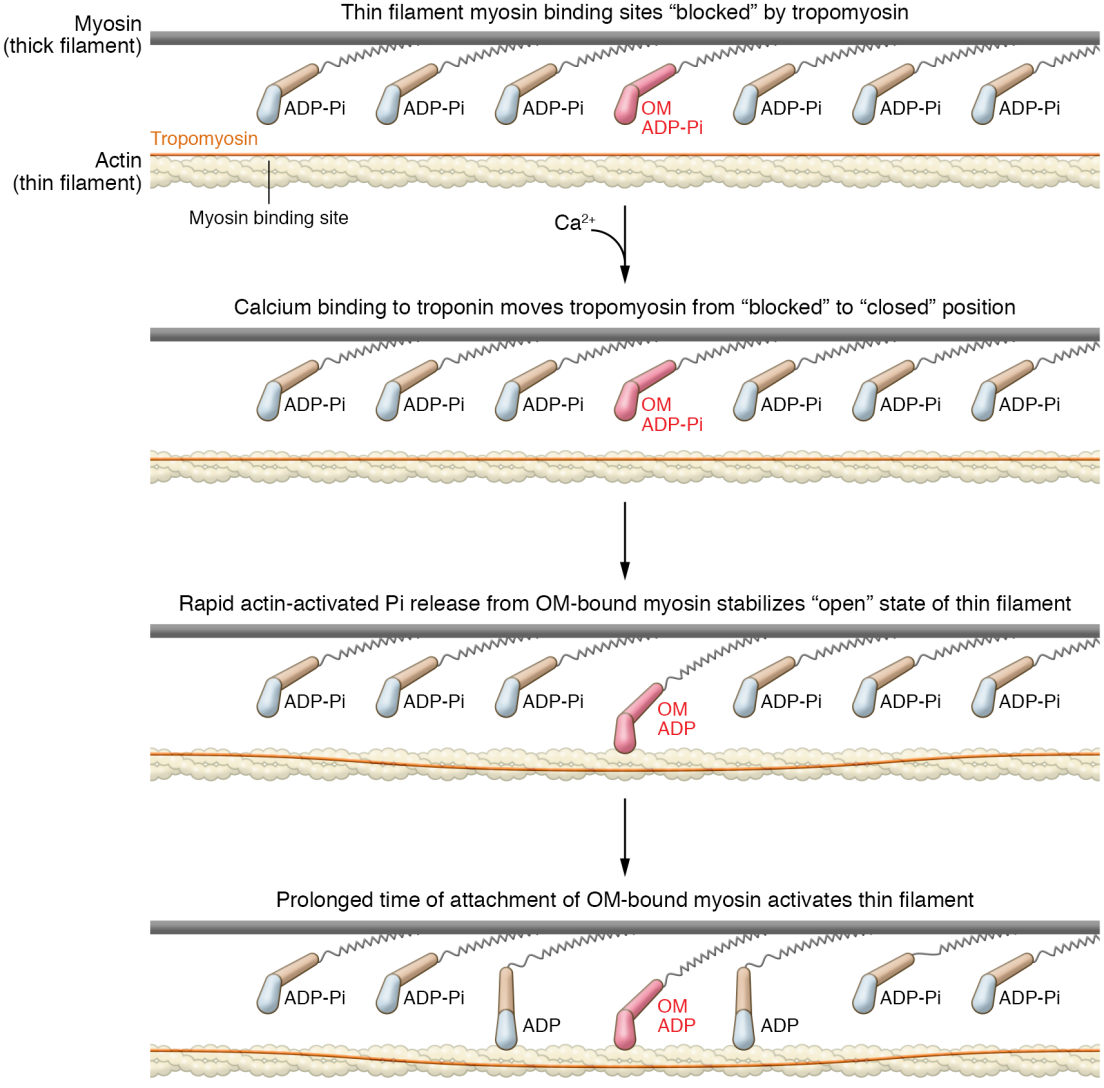
SEPTA model for the mechanism of action of OM. The regulated thin filament is diagrammed, with the myosin binding sites blocked by tropomyosin (blue line) in the absence of calcium. Calcium shifts the position of tropomyosin from a “blocked” state to a “closed” state. While in the closed state, the tropomyosin occasionally shifts to reveal the “open” state of the thin filament. Rapid actin binding and P_i_ release by OM-bound myosin stabilize the open state of the thin filament. Although OM-bound myosin has an inhibited working stroke, its prolonged time of actin attachment keeps the thin filament in the open state, allowing non-OM-bound myosins to attach and undergo uninhibited working strokes. For clarity, myosin molecules are illustrated with single motor domains.

Woody et al. (28) proposed a Stroke Eliminated, Prolonged Time of Attachment (SEPTA) model for the activation of muscle by OM. The SEPTA model suggests that the observed increase in cardiomyocyte force production in the presence of sub-micromolar OM concentrations is due to prolonged actin attachment increasing thin filament activation at sub-saturating calcium concentrations (Figure 2). It has been known since the 1970s that when myosin motors strongly bind to troponin/tropomyosin-regulated actin filaments they cooperatively activate the regulatory machinery (35), i.e., these motors turn the filament on at lower calcium concentrations. Even strongly bound myosins that have had their ATPase activity inhibited by covalent modification of N-ethylmaleimide can activate the thin filament (36, 37). Along these lines, the SEPTA model proposes that long-lived, OM-bound myosins activate the thin filament regulatory system, allowing the OM-free, fully functioning myosin to bind to actin at low calcium concentrations (20, 28). This results in higher-force myocyte contractions at a given calcium concentration than would be achieved in the absence of OM.

A prediction of the original myosin-activating model for OM function suggests that the activation of force will increase with increasing OM as a result of the increase in the rate of phosphate release (step 5, Figure 1), resulting in the increased steady-state population of the force-bearing AM-ADP states (5, 28). However, this is not what has been observed experimentally. Rather, a biphasic response to increasing OM has been observed (22, 25), where the drug inhibits contractility at higher concentration. In contrast, the SEPTA model predicts this biphasic behavior with increasing OM (28). In further support of the SEPTA model, it has been experimentally demonstrated that OM does not increase force production at calcium concentrations that fully activate the thin filament, which is at odds with the originally proposed model, but in agreement with this revised model (22, 24, 26).

Independently of the SEPTA model, OM has been proposed to affect thick filament activation (25), which may explain the decreased cooperativity in isometric force–pCa curves in the presence of OM, which is not reproduced in SEPTA model simulations (28). Interestingly, recent work with rabbit soleus muscle, which expresses MYH7, suggests that inclusion of orthophosphate reverses some of the OM-induced force depression by displacing the drug after Pi release (26). Further experiments will need to be performed to further clarify this orthophosphate effect in cardiac muscle.

Although the mechanism of OM function is different from that originally proposed, it is a small molecule that binds myosin directly and shows therapeutic benefits. Thus, it is clear that myosin is a viable drug target, and that there are opportunities for OM derivatives to be useful for treating other myosin-based diseases.

A recombinant human myosin bearing a disease-causing mutation in MYH7, Arg712Leu (38), has been shown in single-molecule optical trapping experiments to have a defective working stroke, which inhibits motor activity despite near-normal ATPase properties (16). Remarkably, high concentrations of OM rescued motor activity to near wild-type levels in this in vitro experiment (16). The concentration of OM required for rescue (>10 μM) is too high to be therapeutically useful, as this concentration of OM substantially inhibits MYH7. In a second example, OM was also shown to rescue the motility of unconventional myosin, MYO6, with an Asp197Tyr mutation that is associated with deafness in humans and mice (39). Again, the concentration of OM required for rescue of this myosin is too high to be therapeutically useful. Nevertheless, these two studies suggest that it might be possible to target myosin isoform–specific drugs to overcome defects in mechanochemical activity.

## Clinical trials of omecamtiv

OM was first tested for efficacy in a phase II study called COSMIC-HF, in which patients with systolic heart failure were randomized to fixed-dose OM, pharmacokinetic-titrated-dose OM, or placebo for 20 weeks (34). Patients receiving OM demonstrated longer systolic ejection times, higher stroke volumes, smaller LV end-diastolic and end-systolic diameters, and lower levels of N-terminal B-type natriuretic peptide (NT-proBNP). These improvements in cardiac function and remodeling were most pronounced in the pharmacokinetic-titrated group, in which higher mean drug plasma concentrations were achieved. Following these promising results, a global double-blind, placebo-controlled phase III trial, GALACTIC-HF, randomized more than 8000 patients with symptomatic systolic heart failure to OM (pharmacokinetic-titrated) or placebo for a median of 21 months (33). The primary endpoint, a composite of heart failure events or death, was met in 37% of the OM group and 39.1% of the placebo group, with a hazard ratio of 0.92 (95% CI 0.86–0.99, P = 0.03). There was no significant difference in mortality between the groups, but patients receiving OM were less symptomatic and had 10% lower NT-proBNP levels compared with patients receiving placebo. Importantly, there was no increase in cardiac ischemic or arrhythmic events. A second phase III trial (METEORIC-HF) is under way, testing the efficacy of OM to improve exercise capacity using cardiopulmonary exercise testing in patients with systolic heart failure and impaired exercise tolerance (Table 1).

While the results from GALACTIC-HF were favorable for a treatment benefit of OM, the effect size was relatively modest, with an absolute risk reduction of 2% and relative risk reduction of 8% conferred by OM for the primary composite primary outcome. A prespecified subgroup analysis showed that the treatment benefit of OM was accentuated in patients with lower ejection fractions: the relative risk reduction was 17% and the absolute risk reduction was 7.4 events per 100 patient-years with OM versus placebo in patients with ejection fraction ≤22% (40). Similarly, among patients with severe heart failure (New York Heart Association class III–IV), those taking OM experienced a 20% relative risk reduction and an absolute risk reduction of 8.3 events per 100 patient-years compared with those taking placebo (41). Together these data suggest that OM confers the greatest therapeutic benefit, on top of standard neurohormonal and adrenergic blockade, in patients with more severe systolic heart failure. These patients often have limited treatment options and are frequently intolerant of standard heart failure medications because of hemodynamic effects that lower blood pressure and heart rate. Therefore, this is a population urgently in need of treatment alternatives, and OM may fill this need for many patients.

## Rationale for the development of mavacamten

Since the original definition of HCM as “unexplained left ventricular hypertrophy,” the complex functional consequences of a significantly enlarged LV wall coupled to a decreased LV chamber size have dominated the clinical description of the disorder as a disease of “hypercontractility” — a characterization that continues to this day. This serves as the focus for treatment approaches (both existing and in development), with the caveat that, in patients, contractility is approximated by a two-dimensional echocardiography–derived measure of ejection fraction that is inversely proportional to chamber size and is not necessarily an indicator of intrinsic LV contractile properties. Thus, the complexity of the organ-level dysfunction is a significant contributor to the near-protean variability of the clinical disorder and presents an enduring challenge to the design of modern therapeutics. In the obstructive form of HCM, negative inotropes are a mainstay of symptom management (42). These drugs include beta blockers and non-dihydropyridine calcium channel blockers, as well as disopyramide. While classified as a 1A antiarrhythmic sodium channel blocker, the mechanism of action of disopyramide has recently been shown to be more pleiotropic (43). Disopyramide was shown to inhibit peak and late Na+, Ca2+, and K+ currents, with the net effect of shortening action potential duration in human cardiac myocytes isolated from human HCM septal myectomy specimens, without directly affecting myofilament force.

The efficacy of negative inotropic agents in treating outflow tract obstruction in HCM underlies the basic premise that directly manipulating the power output of cardiac muscle at the molecular level via a targeted small-molecule inhibitor of myosin would abrogate the elevated contractile force of the HCM heart and potentially improve symptoms and alter the course of disease progression. A fundamental concept that underlies cardiac muscle contractility is power output, defined as the product of force and contractile velocity (44). At the sarcomeric level, the force parameter is defined as ensemble force (Fens) and represents the product of the force generated by a single myosin head (Funi); the duty ratio, i.e., the fraction of the cycle in which myosin is bound to actin [r(F)]; and the number of myosin heads available to interact with actin (N). As noted in the prior section describing the myosin ATPase duty cycle (Figure 1), these parameters are experimentally addressable at multiple levels of experimental resolution, forming the kinetic and structural basis for the design and characterization of the first-in-class myosin modulator designed to decrease contractile force, mavacamten (originally MYK-461).

Working from the premise that an increase in sarcomere power is a primary molecular cause of HCM, the developers of mavacamten designed a small-molecule screen to identify compounds that bind MYH7 and reduce the maximal actin-activated ATPase rate in bovine myofibrils (8). The expectation was that this reduction would represent an increase in overall total cycling time and thus a decrease in Fens, leading to a decrease in power. Note that these outcomes are, not surprisingly, the opposite of those proposed for the development of OM. Like OM, a concomitant goal was to avoid altering myocellular Ca2+ homeostasis. Initial characterization of the identified lead compound (MYK-461) was carried out in multiple systems representing varying levels of biological complexity. Measurement of steady-state ATPase activity in both murine cardiac myofibrils and isolated bovine S1 after treatment with mavacamten demonstrated potent dose-dependent decreases and an IC50 of 0.3 μM, while transient kinetics revealed a dose-dependent decrease in Pi release without an effect on ADP release, consistent with a decrease in total cycling rate (steps 5 and 6, Figure 1). Finally, mice carrying known myosin-linked HCM mutations (Arg403Gln, Arg453Cys) treated with mavacamten before the development of LVH exhibited significant decreases in cardiac fractional shortening and LV wall thickness and an overall decrease in LV fibrosis as compared with WT controls (8). Notably, treatment with mavacamten after the onset of pathogenic remodeling did not alter LV fibrotic burden in either model. A study by Kawas et al. used a series of steady-state and pre-steady-state kinetic approaches across systems of varying complexity and different species to further refine the effect of mavacamten on the myosin mechanochemical cycle (45). Their observation that mavacamten slowed actin sliding velocities in the in vitro motility assay, coupled with the prior steady-state ATPase kinetics results, was consistent with a more complex mechanistic effect on the myosin cycle beyond simply a decrease in the rate of Pi release. The authors suggested that mavacamten effectively “removes” myosin heads from the ATPase cycle, decreasing Fens by decreasing N (Figure 1). Taken together, these studies expanded the focus of the field from the characterization of a novel small-molecule myosin modulator to questions regarding fundamental biochemical properties of cardiac myosin and even potential mechanisms for the pathogenesis of HCM.

## Mavacamten as a stabilizer of myosin’s super-relaxed state

The concept of modulating the generation of force in muscle via modulating the accessibility of myosin heads is not, by itself, novel. For example, a central component of the Frank-Starling effect is an increase in actin-myosin cross-bridge formation via mechanical stretch. Over the past decade, an array of studies has begun to characterize the potential role of equilibrium shifts in myosin structure and dynamics as an important regulator of function. We focus here on the proposed effects of mavacamten and important unknowns (46, 47).

A 2010 study by Stewart et al. identified a state of myosin with very slow ATPase activity in relaxed, permeabilized skeletal muscle. This super-relaxed (SRX) and sequestered state of myosin was postulated to play a role in muscle thermogenesis (48). Subsequent studies revealed that the properties of the SRX state were distinct between cardiac and skeletal muscle, specifically with respect to the lack of a cooperative mechanism for recruitment into the active cycling pool in cardiac muscle (49). The current theory regarding the steady-state distribution of myosins proposes three general states defined by both structure and ATP turnover rate (listed in decreasing rate order): actin-activated; disordered, relaxed (DRX); and SRX. It has been proposed that the myosin SRX state forms the structurally distinct interacting-heads motif (IHM) of myosin in the thick filament, where the myosin motors interact with each other and fold back against the S2 fragment, similar to the off state of smooth muscle myosin (50).

**Figure 3:**
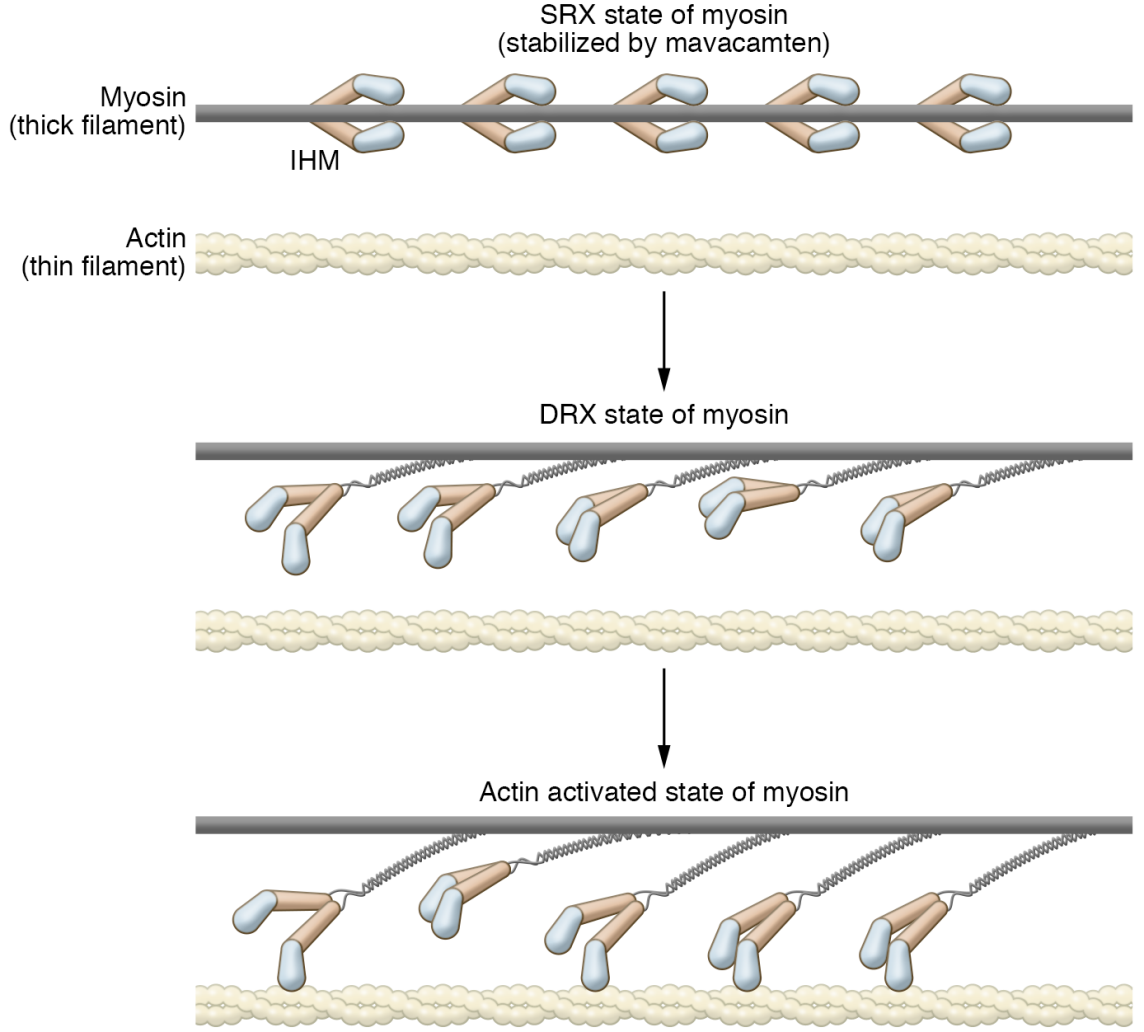
A hypothetical model of the relationships between SRX, DRX, and actin-activated states of myosin and effects of drugs. The three states of myosin — SRX, DRX, and actin-activated — are diagrammed. The motor domains in a myosin molecule are proposed to interact with each other and the thick filament to form the IHM in the SRX state. While in the SRX state, the myosins are not available to interact with actin, and they are not involved in muscle contractility. Mavacamten is proposed to stabilize the SRX state. Myosins in the DRX state do not interact with the thick filament and are available to bind to actin in response to thin filament activation. Myosins in the actin-activated state bind to actin and undergo their mechanochemical cycle as outlined in Figure 1.

Two recent studies provided insight into the biochemical and structural mechanisms of the SRX-DRX transition and its relationship to mavacamten. Rohde et al. used a purified bovine cardiac myosin fragment approach where they compared ATP turnover of single-headed (S1) fragments and double-headed heavy meromyosin (HMM) under varying conditions (basal and actin-activated) in the presence and absence of mavacamten (51). Their results pointed to the presence of an autoinhibited, SRX state for HMM, but not for S1. The SRX state of HMM was stabilized by the binding of mavacamten. In a complementary study, Anderson et al. measured the percentage of myosin heads in the SRX state in an engineered human β-myosin S1 construct and two HMM constructs that contain either the first 2 or first 25 heptad repeats from the S2 region, with the 25-heptad construct being predicted to form the IHM, but not the 2-heptad construct (9). The percentage of heads in the SRX state was measured by single-turnover kinetic experiments at varying ionic strengths. The 25-heptad HMM construct had substantially more heads in SRX than the other two constructs. Additionally, the fraction of 25-heptad heads in SRX decreased with ionic strength, supporting the model that the SRX state correlated with the IHM. Similar results had been obtained by Rohde et al. in their purified myofibril system (51). Finally, single-turnover kinetic measurements indicated that mavacamten binding led to a large increase in the SRX state of the long-heptad HMM and the number of molecules with heads in a folded-back state as determined by electron microscopy (9). However, the folded-back state they observed may or may not be the IHM state. Thus, while many of the structural and kinetic details of the relationships and transitions among the myosin DRX, SRX, and IHM states have yet to be fully resolved, considerable progress has been made in our understanding of the molecular mechanism of mavacamten to preferentially stabilize the SRX state of myosin, shifting the equilibrium toward the off (and possibly the IHM) state (Figure 3).

The observed potent SRX-stabilizing effects of mavacamten across a broad range of experimental systems, from purified HMM to isolated myofibrils and intact cells/tissues, have provided insight that will continue to advance our understanding of the role of these basic biological properties in health and disease. There are limitations to the current approaches, in particular the lack of a rigorously quantitative measure of the kinetic transition from the SRX state and the need for a better high-resolution understanding of the structural basis of these dynamic states. The former is particularly important in the context of the more recent observations and hypotheses regarding the potential causal role of a destabilized SRX state in HCM. For example, the electrostatic landscape of myosin is disrupted by point mutations (the myosin mesa) or haploinsufficiency of myosin-binding protein C (MYBPC), causing a change in the spatial distribution of SRX (9, 44, 52–54). HCM is defined by its near-protean phenotypic variability, including non-obstructive forms with varying degrees of hypertrophic remodeling. Without a more rigorous, high-resolution quantitative measure of SRX destabilization and a better understanding of the native regulation of the DRX-SRX-IHM states, it will be difficult to account for the broad spectrum in the magnitude of cardiac remodeling and outcomes on the basis of this proposed disease mechanism. However, irrespective of the mechanistic details, mavacamten, and, more recently, aficamten (CK-274) (10), are poised to have a powerful impact on both basic biological insights and patient care in HCM.

## Clinical trials of mavacamten and aficamten

Mavacamten was first tested for efficacy in an open-label, nonrandomized, phase II trial of patients with HCM and LV outflow tract obstruction (55). The trial met the primary endpoint, which was a significant reduction in exercise-induced LV outflow tract gradients in the higher-dosed cohort (103 ± 50 to 19 ± 13 mmHg, P = 0.008), as well as secondary endpoints of improvements in exercise capacity (peak VO2) and dyspnea scores from baseline. Following these results, a double-blind, randomized, placebo-controlled phase III trial tested the efficacy of mavacamten in 251 patients with HCM and symptomatic LV outflow tract obstruction (EXPLORER-HCM; ref. 56). This trial also met its primary efficacy endpoint, with mavacamten conferring a significant improvement in peak VO2 and New York Heart Association symptomatic classification over placebo (37% vs. 17% for the combined endpoint, P = 0.0005). Treatment with mavacamten also favorably impacted cardiac remodeling with reductions in LV mass and left atrial volumes (57). The drug has been well tolerated, with only a few subjects experiencing transient and reversible reductions in LV ejection fraction below 50%. Based on these results, mavacamten is under consideration by the FDA for approval to treat patients with symptomatic obstructive HCM. A randomized phase III trial of mavacamten (VALOR-HCM) that enrolled patients with HCM and severe symptomatic outflow tract obstruction referred for septal reduction therapy was also just completed (58).

Aficamten is a next-generation cardiac myosin modulator that has a similar mechanism of action to mavacamten, but has a shorter half-life and a shallower concentration-response profile (10). Preliminary results from a recently completed phase II trial of aficamten (REDWOOD-HCM) showed reductions in LV outflow tract obstruction and symptomatic improvement within 2 weeks of drug initiation. Results from a recently completed trial of aficamten in patients with symptomatic obstruction despite being on disopyramide are pending, and a phase III trial of aficamten in obstructive HCM is in the planning phase (Table 1).

Given the promising results described above, it seems likely that myosin modulators will become one of several options for treating patients with symptomatic LV outflow tract obstruction. However, it remains to be determined whether they will be efficacious in improving outcomes in patients with symptomatic non-obstructive HCM — a cohort in whom several prior trials of other pharmaceutical agents have failed to show a treatment benefit (59–61). In a double-blind, placebo-controlled phase II trial of mavacamten in 60 symptomatic patients with HCM without LV outflow tract obstruction (MAVERICK-HCM), mavacamten did not significantly improve exercise capacity or symptoms compared with placebo (62). However, circulating levels of biomarkers N-terminal pro–brain natriuretic peptide and cardiac troponin I were reduced, suggesting a decrease in myocardial wall stress. Based on these results, a larger and longer phase III trial of mavacamten, as well as a phase II trial of aficamten, for patients with non-obstructive HCM are currently in the planning phase. A pilot phase IIa study of the safety and preliminary efficacy of mavacamten in patients with heart failure with preserved ejection fraction is also under way (Table 1).

## Key considerations for patient selection and treatment responses

With myosin modulators poised to move from clinical trials to clinical practice pending FDA approval of OM and mavacamten, there will be a number of factors to balance in selecting patients in whom to initiate therapy. For OM, the prespecified subgroup analysis of the GALACTIC-HF trial suggests that patients with more severe systolic heart failure, particularly those who are intolerant of neurohormonal or adrenergic blockade, would be a very reasonable initial target population. For mavacamten, the initial target population will be patients with HCM with symptomatic outflow tract obstruction refractory to beta and calcium channel blockers. In which subgroups mavacamten will confer a significant treatment response, and potentially obviate the need for septal reduction therapy, still remains to be determined. In EXPLORER-HCM, despite the overall highly positive effects in comparison with placebo, 50% of patients on mavacamten still had some residual symptoms, and 25% had residual LV outflow gradients of more than 50 mmHg after exercise. The mechanisms of outflow tract obstruction are complex, and supranormal ejection fraction is a frequent, but not the sole, component. For example, it is possible that patients with massive hypertrophy, or in whom the contribution of mitral valve and subvalvular abnormalities to obstruction is substantial, may derive less benefit from mavacamten and still require surgical intervention. Other patients may choose septal reduction therapy over potentially lifelong medical therapy, particularly those who are young and/or low-risk surgical candidates. For women of childbearing potential, interruptions in myosin modulator therapy could be problematic during pregnancy and nursing.

A longer-term objective for mavacamten or aficamten beyond alleviating obstruction is the potential for disease modification. Even with complete surgical relief of obstruction, peak VO2 fails to improve in approximately 40% of patients (63), which supports the findings that diastolic dysfunction and factors other than obstruction are the primary determinants of exercise capacity in HCM (64, 65). Mavacamten decreases diastolic stiffness and improves lusitropy in human engineered heart tissue (66). These properties, combined with the theoretical reduction in energetic demand by reduction of maximum power output, would be expected to favorably impact the fundamental deficits that drive disease progression in HCM. This is particularly relevant for patients with symptomatic non-obstructive HCM or heart failure with preserved ejection fraction, for whom few treatment options are available. Trial designs and eligibility criteria will need to be carefully considered to maximize the chances of showing a treatment benefit of mavacamten or aficamten within highly heterogeneous patient cohorts.

An important consideration for the clinical implementation of all myosin modulators is the potential for sex- or race/ethnicity-specific effects. Women and racial minorities are underrepresented in all trials to date, not dissimilarly to other trials in heart failure and cardiology in general. Cost and access to care at specialized centers with experience prescribing these drugs will also need to be systematically addressed. Finally, it remains to be seen whether genetic etiology will be a determinant of treatment responses for patients with HCM or DCM. Genotyping is now standard in HCM trials, but is completely absent from any clinical trials of systolic heart failure, despite the fact that 30%–40% of genotyped cohorts of nonischemic DCM have a Mendelian genetic basis.

## Conclusions and future directions

Although we are in the early days of development, the studies described above indicate that modulation of cardiac function by myosin-binding drugs is a strategy that holds great promise for treating a spectrum of cardiomyopathic disease and heart failure. It is important to note that MYH7-binding agents also have the potential to affect the activities of MYH7 expressed in skeletal muscle (26, 67), so there are additional therapeutic opportunities as well as off-target effects to be considered that have not been directly studied in patients. The known actions of these drugs in the heart include both the predicted modulation of the mechanochemistry and regulatory state of myosin, and unexpected effects, such as activation of the thin filament by OM, that could lead to novel clinical applications and treatment algorithms. Combinations with other drugs with complementary mechanisms of action hold substantial potential for improving clinical efficacy. For example, thin filament activation conferred by OM suggests that low doses of calcium-sensitizing inotropic agents could have additive effects to enhance contractility in systolic heart failure. With FDA approval imminent for the first-in-class myosin modulators, predictors of treatment benefit for different subgroups of patients should soon become evident. Results from ongoing and planned trials, as well as real-world clinical experience with these drugs, are therefore highly anticipated.

## Figures and Tables

**Table 1:**
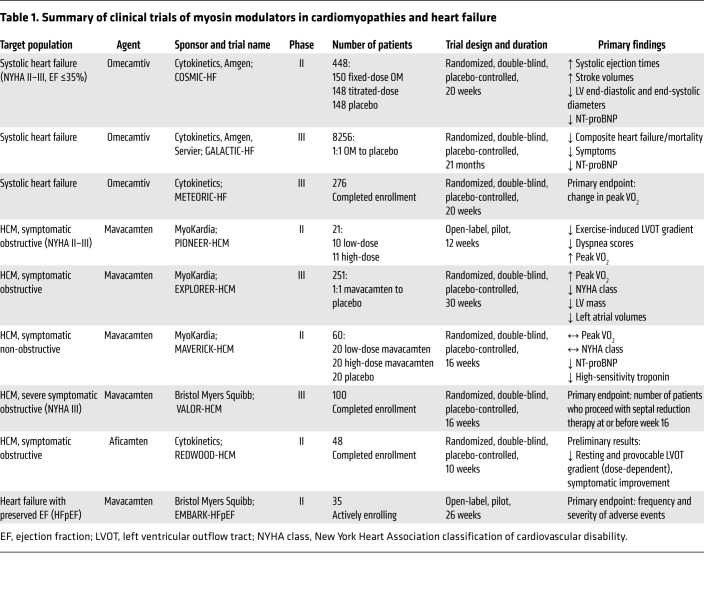
Summary of clinical trials of myosin modulators in cardiomyopathies and heart failure

## Version 1 03/01/2022

Electronic publication
